# Measurement Uncertainty Estimation in Amperometric Sensors: A Tutorial Review

**DOI:** 10.3390/s100504430

**Published:** 2010-04-30

**Authors:** Irja Helm, Lauri Jalukse, Ivo Leito

**Affiliations:** Institute of Chemistry, University of Tartu, 14a Ravila str. 50411 Tartu, Estonia; E-Mails: lauri.jalukse@ut.ee (L.J.); irx@ut.ee (I.H.)

**Keywords:** amperometric sensors, measurement uncertainty, uncertainty sources

## Abstract

This tutorial focuses on measurement uncertainty estimation in amperometric sensors (both for liquid and gas-phase measurements). The main uncertainty sources are reviewed and their contributions are discussed with relation to the principles of operation of the sensors, measurement conditions and properties of the measured samples. The discussion is illustrated by case studies based on the two major approaches for uncertainty evaluation–the ISO GUM modeling approach and the Nordtest approach. This tutorial is expected to be of interest to workers in different fields of science who use measurements with amperometric sensors and need to evaluate the uncertainty of the obtained results but are new to the concept of measurement uncertainty. The tutorial is also expected to be educative in order to make measurement results more accurate.

## 1. Introduction

Amperometric sensors are applied widely to the concentration measurements of different analytes, e.g., gas components in the gas phase or analytes dissolved in a liquid medium. They offer good sensitivity [1,2] and wide linear range [3]. They can be low-cost and can be mass produced via microfabrication technology [4]. They are simple to use and are widely used in different areas of chemical analysis such as environmental monitoring, surveillance, security, industrial safety and medical and health applications. Numerous substances can be determined with amperometric sensors [1,5] (see Table 1 for an overview).

Uncertainty estimation of measurement results (including chemical analysis results [6]) has nowadays become a standard requirement [7,8]. Results without an uncertainty estimate cannot be considered complete [9]. At the same time, due to the nature of chemical measurements estimation of their uncertainty is often complicated [7]. This has resulted in a still continuing mismatch between the requirements imposed on laboratories and their ability to meet them.

The aim of this tutorial is to give an overview of the main uncertainty sources that influence measurements with amperometric sensors, briefly look at two major approaches for uncertainty estimation and to illustrate practical uncertainty evaluation with two case studies. Our emphasis is on depth and usefulness for potential readers rather than on a (formally) exhaustive coverage of literature, therefore the literature references are selective rather than extensive. This tutorial is expected to be of interest to workers in different fields of science who use measurements with amperometric sensors and need to evaluate the uncertainty of the obtained results. We focus on low-temperature membrane amperometric sensors based on chemical reactions. High-temperature amperometric sensors as well as biosensors are outside the scope of this review.

## 2. Design and Principle of Operation of Amperometric Sensors

Numerous reviews have been published on design and operation of amperometric sensors [1,5,10–12] and only a brief introduction will be given here. The fundamental process for sensing an analyte by an amperometric sensor can be described in four steps: (1) the analyte diffuses to the sensing electrode. In order to achieve selectivity and/or diffusion-limited working mode this diffusion may proceed through a membrane or some other diffusion barrier. (2) The analyte is adsorbed on the sensing electrode. (3) The electrochemical reaction occurs. (4) The reaction products desorb from the sensing electrode and diffuse away [13,14].

**Scheme 1.**
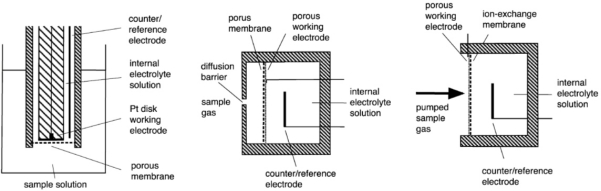
The most frequently used amperometric sensor designs–Clark’s, GDE and SPE sensors (reprinted from [1] with permission from Elsevier).

Amperometric sensors are based on electrochemical cells consisting of working electrode, counter-electrode and reference electrode that are in connection through an electrolyte phase. By the design the sensors can be broadly divided into three groups: Clark type, SPE and GDE, see Scheme 1 (see [1] for more information). On the working electrode the electrochemical reaction involving the analyte is carried out. The response (analytical signal) of the sensor is the current between the working electrode and counter-electrode. The working conditions of the sensor are usually chosen such that the sensor works in the diffusion-limited mode [1,5,10] and the current is independent of the working electrode potential. In this mode the mass-transfer rate of the analyte is slow and the Faradaic current is controlled by diffusion rather than the kinetics of the electrode reaction [5,15]. This assures a linear dependence of the current on concentration of the analyte [1,14]. The diffusion barrier is usually formed by the membrane (Clark, GDE) or is created artificially by a mechanical barrier (SPE) [14].

If the limitation is on the kinetics of the reaction then the response of the sensor is non-linear and the sensor will be more susceptible to ageing [1].

The porous PTFE-membrane of the GDE-devices serves to restrict the transport of the analyte to the electrode, but a further artificial barrier in the form of a covering plate with holes of controlled dimensions is usually still needed to obtain a well defined diffusion control and stable signal. This diffusion barrier also reduces the effects of drafts in the atmosphere being sampled [1].

In the SPE-membrane based sensors, the electrode surface directly faces the sample gas or liquid and therefore essentially no diffusion barrier is present [1,2]. This makes the sensitivity and response time of the SPE sensors better than those of the Clark or GDE sensors [16]. The virtual absence of diffusion layer also greatly reduces the temperature-dependence of the response of a SPE electrode [16]. The negligible diffusion barrier also has a downside. If diffusion is very fast then there is the danger that the sensor will not be operating in the diffusion-limited mode any more resulting in loss of linearity [17]. Therefore in SPE sensors an artificial diffusion barrier is sometimes added. SPE sensors also have a stronger dependence of the signal on the gas flow rate [1,14] and are therefore usually used in systems with a forced and constant gas flow.

The electrolyte phase carries the cell current by enabling the transport of charge carriers in the form of ions and often provides co-reactants to electrode and allows the removal of ionic products from the reaction site. Note, that counter and reference electrodes may be combined into a single electrode [1,5]. Each sensor can have a unique design and a different set of materials and geometries for membranes, electrolytes, and electrodes in order to take advantage of chemical properties of a specific target analyte and survive under various operating conditions [5].

A critical issue in design of amperometric sensors is achieving selectivity, *i.e.*, situation that the sensor current depends on analyte concentration but is insensitive towards possible interferents in the solution. In early amperometric measurements selectivity was achieved by the choice of working electrode material and the potential of the working electrode.

A major breakthrough in this field was achieved in 1953, when Leland C. Clark developed the practically usable membrane oxygen sensor for measuring oxygen tension in the cardiovascular system *in vitro* and *in vivo* [18]. The choice of membrane material became the third important tool for achieving selectivity. With this advancement actually the amperometric sensors were born. After patenting the method in 1959, electrochemical membrane covered amperometric sensors have become a common method *in situ* measurement of oxygen and the design is often called ”Clark type”.

By appropriate selection of the membrane material (PTFE, PFA, FEP, PE, PP, silicone, cellophane *etc*.) and specific properties, one can control the analytical characteristics of the sensor, permitting the analysis of several analytes over a wide range of concentration [5]. Modern Clark electrodes are often fitted with a porous PTFE membrane. Because of the hydrophobicity of the material, the pores are not wetted by the aqueous solution and are impermeable for ions and polar organic compounds but allow the transport of dissolved non-polar gases to the electrode [1]. Out of the gases normally dissolved in the aqueous environment only oxygen can undergo reduction at the working electrode. This way the selectivity of the dissolved oxygen sensor is ensured. Several diffusion layers are formed in the classic Clark sensor: the electrolyte layer, the membrane and a stagnant layer [5,14]. The thinner are the layers the higher the sensitivity and the faster the response.

The electrolyte can be an aqueous or a non-aqueous solution or a so-called solid-phase electrolyte (SPE), which in most cases is a conductive polymer. These are good because of their high boiling point and often very high ionic conductivity. A typical solid polymer electrolyte is Nafion, a hydrated copolymer of poly(tetrafluoroethylene) (PTFE) and polysulfonyl fluoride vinyl ether, which contains sulfonic acid groups. It has more positive features, such as high structural stability and resistance to acids and strong oxidants. The only limitation for field use was the issue of the water in the Nafion freezing at low temperatures. [5].

The second breakthrough in amperometric sensor design–introduction of nanostructured materials – has taken place during the turn of the century [3,19–21]. It is clear that sensor’s sensitivity depends on the surface area on which the electrochemical reaction takes place. The limiting current is proportional to the electrochemically active surface area, *i.e.*, the three-phase boundary (TPB: interface between the gas, the electrode and the electrolyte) area, because the electrochemical reaction takes place only in this area [22]. Nanostructures can dramatically increase the three-phase boundary area, followed by an enhancement of the sensors sensitivity [5]. A further improvement of the sensitivity from ppm to ppb gas concentration levels can be obtained using the new membrane–electrode assembly composed of a PTFE membrane and nanocomposite materials of carbon nanotubes and PTFE [5]. In contrast to the Clark electrode the sensors of this type are not affected by evaporation of water because the porous electrode is directly in contact with the bulk of the electrolyte solution. The mass transfer of the analyte from the sample to the working electrode can be faster, resulting in shorter response times and higher currents that leads to higher sensitivity [5]. Newer design concepts have also been proposed, so that new breakthroughs are to be expected [23–25]

## 3. General Principle of Amperometric Measurement in Gas Phase and in Liquid Phase

Amperometric sensors measure the chemical potential of the analyte in the gas phase (termed as fugacity) and/or in the liquid phase (termed as activity) [26]. Thermodynamically, gas-liquid phase equilibrium is described by the Henry’s law as:

(1) f=K·a

where *f* is the fugacity of the analyte in the gas phase, *K* is an equilibrium coefficient for a particular analyte and liquid, called Henry’s constant [15,27] and *a* is the activity of the analyte in the liquid phase. For low-pressure systems the fugacity of the analyte in the gas phase can be assumed to be equal to its partial pressure and the liquid-phase activity of the analyte can be expressed as product of activity coefficient and concentration of analyte, thus:

(2) p=K·γ·C

where, *p* is the partial pressure of the analyte, γ is the activity coefficient of the analyte and *C* is the concentration of the analyte. According to this equation the solubility of the analyte is proportional to its partial pressure above the liquid phase. The gases that have low boiling points and lack the reactivity towards water (H_2_, O_2_, CO, *etc*.) have low solubility in water and the temperature-dependence of solubility is linear. Gases that react with water (NH_3_, SO_2_, CO_2_, *etc*.) have higher solubilities. It is often assumed that the activity coefficient is equal to 1.0 or at least constant. However, in real samples this assumption often breaks down [28]. Especially important from practical point of view are different salt solutions, such as e.g., sewage [28] and seawater [26,29]. Using Fick’s first law and Faraday’s law the following general expression for the steady state current of an amperometric sensor can be written [15,17]:

(3) $$I=\frac{n\cdot F}{R_{k}+\sum_{i=1}^{3}R_{i}}\cdot p$$

where *I* is the sensor output current, *n* is number of electrons involved in electrochemical reaction, *F* is the Faraday constant, *R*_k_ is the kinetic resistance of the electrochemical reaction and *R*_i_ is the analyte diffusion layer resistance of a layer i. This generic equation holds for Clark, GDE and with some reservations also for SPE sensors. Albantov and Levin regard the diffusion layers mathematically as a set of resistors sequentially connected. The resistivity of a layer *i* to diffusion is described by the following equation for membrane-covered sensors [15]:

(4) $$R_{i}=\frac{\delta_{i}}{S_{i}\cdot D_{i}\cdot A_{i}}$$

where, *δ*_i_ is the thickness of the diffusion layer i, *S*_i_ is analyte solubility in the diffusion layer i and *D*_i_ is analyte diffusion coefficient in the diffusion layer i, *A* is the diffusion layer area (projected area of the electroactive surface in Clark type sensors [10]). The construction parameters of the sensor, such as surface area of the vacuum-deposited metal layer, width of the measurement cell, thickness of the metal layer, membrane thickness, membrane character (number of ionic groups in the total mass of polymer), the way of analyte supply to the working electrode surface (axial or radial) and composition of the internal electrolyte impose have an impact on the sensor signal. In reference [14] a comprehensive mathematical model is given, which describes the effects of these variables.

Equation 3 is the general measurement model of the amperometric sensors. In the classical Clark sensors in the gas phase usually two diffusion layers are assumed [30] and in the liquid phase three diffusion layers [15,31]. In the GDE and SPE sensors the diffusion limitation can be achieved using mechanical barriers and a single diffusion layer is used in eq (3) [32].

## 4. Literature Survey: Overview

Because of the nature of amperometric measurement described in the previous section it is clear that it is affected by numerous uncertainty sources. In the literature there is no shortage of reports that describe amperometric sensors of different design and address their characteristics, such as response time, detection limit, linearity, repeatability and sensitivity of sensors [14,16,22,33–43]. There are also works that discuss accuracy [38,44] and drift [34,36,37]. But there are only a handful of papers that describe combined uncertainty (*i.e.*, uncertainty taking into account all relevant uncertainty sources) estimation of amperometric measurement results and analyze the relevant uncertainty sources. Most of them are devoted to amperometric oxygen sensors [31,45–48]. Jalukse and Leito have carried out in-depth analysis and modeling of amperometric dissolved oxygen sensors. They identified 16 separate uncertainty sources [31] and found that the relative expanded uncertainties (*k* = 2) obtained by experts under laboratory conditions varied between 1% and 9%. More recently Nei has stated that uncertainties in amperometric dissolved oxygen measurement at field laboratory level tend to be larger (between 5% and 20%) [46].

Table 1 gives the overview of the articles found that address at least three uncertainty sources in amperometric sensors. The uncertainty sources will be reviewed in detail in the next section. The general observation from the literature survey is that, apart from certain articles on dissolved oxygen concentration measurement, uncertainty of measurement results obtained with amperometric sensors is rarely discussed in the literature. Most authors examine certain characteristics that are relevant to uncertainty but cannot be easily combined into the combined standard uncertainty estimate of the result *u*_c_ (see below for terminology of uncertainty estimation).

## 5. Literature Survey: Sources of Uncertainty

Based on the generalization of the literature survey and the experience from our laboratory we discuss below the main uncertainty sources relevant to amperometric sensors.

### 5.1. Temperature (Compensation)

The rate of the diffusion or permeation (below diffusion) processes is influences by temperature. Temperature directly influences the following: widths of the diffusion layers, diffusion coefficients of the analytes in the different layers [29,62–65]. This is the reason why amperometric sensors are normally equipped with accurate temperature measurement capability and the results are in most cases corrected for taking into account the difference between measurement and calibration temperatures. In most cases measurement and calibration are carried out at temperatures that differ from each other (at least by few degrees). This temperature difference has to be taken into account. Temperature effects cause uncertainty in amperometric measurement via two factors: (1) limited accuracy of temperature measurement (during calibration and measurement) and (2) limited accuracy of compensation for temperature difference between calibration and measurement [31]. The latter may in turn be due to inaccuracy of the model underlying the temperature compensation or the inaccuracy of the value(s) of the parameter(s) involved in the compensation [31].

This component is also influenced by changes in properties of diffusion layers: for example: deformation, ageing and contamination of the membrane in the case of membrane-based electrodes. Different sensor designs are affected somewhat differently by temperature. In the Clark and GDE type sensors temperature affects the permeability of the membrane (activation energy of diffusion through membrane). At the same time in the SPE sensors the analyte does not need to diffuse through the membrane. The temperature dependence of permeability is determined by measurement of sensor current at different temperatures at constant (and known) analyte concentration. The temperature dependence can then be either used empirically [52,54,56,57,66] or converted into activation energies of diffusion through the diffusion layers [10,31]. The importance of this uncertainty source is widely acknowledged, but rigorous evaluation of magnitude of this uncertainty is generally not done (except in reference [31]).

### 5.2. Drift

Drift is defined by VIM [67] as continuous or incremental change over time in indication, due to changes in metrological properties of a measuring instrument. Instrumental drift is related neither to a change in a quantity being measured nor to a change of any recognized influence quantity. In amperometric sensors all changes during sensor use, which lead to changes of the sensor properties compared to the time of calibration cause drift. Possible causes of drift are unstable reference potential, caused by contamination, poisoning or consumption (depletion) of the reference or counter-electrode, local changes of electrolyte concentration and/or pH, contamination or poisoning of the working electrode (changed catalytic activity) [10,29,68]. One of the most important of them is change of the diffusion layer that limits the mass transfer [31]. Changes in the properties of the working electrode do not affect the sensor as long as mass transfer remains the rate-limiting step (Clark and GDE type sensors). However, in more severe cases the sensor may begin to work in a mixed kinetics mode, leading to drift of the parameters and loss of linearity. The factors causing drift are known to change with age and periodically over time. Some factors can be predicted while others are more or less random. In order to evaluate the uncertainty due to drift the sensor signal has to be monitored in time at constant analyte concentration. This can be done either continuously or periodically. Keeping the measurement conditions constant is very important.

### 5.3. Stirring Speed or Flow Rate

**Figure 1.**
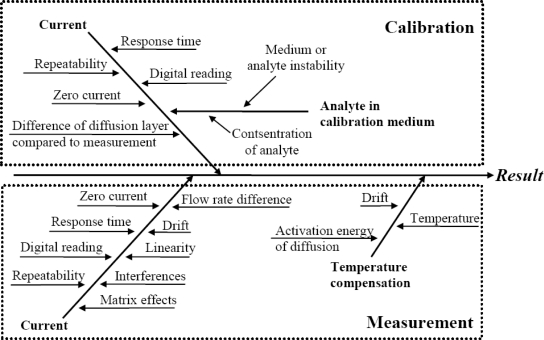
Uncertainty sources in amperometric measurement.

Stirring speed or solution (or gas stream) flow rate (below termed flow rate) influences the result, because it affects the thickness of the outer diffusion layer. This happens because of analyte consumption by the sensor at the boundary layer between the sample and the membrane [15,29,31]. If the measurement is carried out in the gas phase then the dependence of the sensor signal on the flow rate strongly depends on the sensor type and design. The higher is the porosity of the contact between the sensor and the measured solution (*i.e.*, membrane in the case of Clark and GDE sensors and working electrode in the case of SPE sensors) the higher is the sensitivity of the sensor signal towards flow rate [1,14]. At low values of gas or liquid flow rate the signal dependence on the flow rate is almost linear. Above a certain value the linearity is lost and if the flow rate is further increased then the influence of flow rate on sensor signal becomes negligible. This is because the stagnant layer thickness decreases with increasing volumetric flow rate and stops playing a significant role in the overall diffusion resistance [14]. When measuring in solution then a stagnant solution layer always forms on the membrane surface [15]. This layer decreases the signal, compared to the respective signal in the gas phase (with equal activity of the analyte) [31]. If calibration and measurement are carried out at different flow rates then this effect has to be corrected for or taken into account as an uncertainty source (see Figure 1, difference of diffusion layer compared to measurement) [31]. The thickness of this stagnant layer strongly depends on the flow rate [29]. If the flow rate is the same during calibration and measurement (e.g., if both are carried out in the same measurement cell) then the flow rate uncertainty component need not be taken into account. The effect of flow rate on the sensor response can be evaluated by carrying out measurements at constant temperature and constant analyte concentration and varying the flow rate.

### 5.4. Repeatability

Repeatability is measurement precision under repeatability conditions (set of conditions including the same measurement procedure, same operator, same measuring system, same operating conditions and same location, and replicated measurements over a short period of time) of measurement [67]. It is commonly expressed as standard deviation *s*_r_ of the values obtained from repeated measurements.

In the literature many repeatability estimates for different sensors can be found [16,22,33–37]. Sensitivity of the sensor is closely related to repeatability. High sensitivity maximizes signal to noise ratio and thus improves repeatability [69]. In other words, the greater the sensitivity the better is normally the capability of the sensor to distinguish between the signal and the background noise [29]. The stability of the sensor signal and thus the repeatability can be influenced by several parameters, for example fluctuations of measurement temperature, flow rate [31] or reference potential). Repeatability is usually dependent on the magnitude of the signal itself and is often roughly proportional to it. This, however, needs to be experimentally confirmed for a particular sensor and repeatability should therefore be investigated at different concentration levels. Repeatability is also influenced by the flow rate [31]. Therefore, if measurements are carried out at different flow rates then it is necessary to evaluate the dependence of repeatability on stirring speed.

An important parameter related to repeatability is within-laboratory reproducibility (also known as intermediate precision) *s*_R_. It characterizes long-term measurement precision within a laboratory [67,70]. By its nature reproducibility is a compound parameter, accounting for repeatability, as well as all other effects that may have different magnitudes on different days. This involves changes in the sensor due to ageing (drift os sensor characteristics), uncertainty sources related to calibration, *etc.* Only effects that retain their magnitude over a long time period remain outside of within-lab reproducibility.

Within-lab reproducibility is a very useful characteristic because it is relatively easy to determine it experimentally and it is one of the two cornerstones of the validation-data-based uncertainty estimation approach (see below). Literature data on reproducibility of amperometric measurements are scarce [59,60] and in addition it may be possible that in fact repeatability is what is meant in references [59] and [60].

### 5.5. Response Time

Sensor response time is one of the basic quality indexes for evaluating the performance of electrochemical sensors [32]. It is a parameter describing dynamic properties of the sensor with respect to changes of the analyte concentration [14]. Response time of amperometric sensors has been investigated extensively both experimentally [16,22,35,36,38,41–43,59,72] and via theoretical simulations [32,73].

The diffusion process, which controls the response time, can be modeled using Fick’s second law [74,32]. The response time of the sensor is commonly specified by the so called T_90_. This value indicates the time required to reach 90% of the sensor’s stationary current corresponding to the analyte concentration [32,43]. Other values referring to different percents of the stationary current, such as T_63_, T_95_, etc, are also used [14,49,51,75]. Contributing factors include diffusion through the diffusion barriers, membrane(s), electrolyte, and also kinetics of the electrode reaction (where appropriate), as well as some aspects of the electronic circuit [14,32,43]. In the case of GDE and Clarke type sensors the diffusion of analyte through membrane is the most significant factors of these [10]. Since mass transport by diffusion through the membrane is the slowest step in the overall process, dramatically shortened response times can be obtained by using thinner membranes [29].

To model this effect, diffusion transport must be understood and characterized. This is done by measurement of the sensor output signal in time, first without and then with and finally again without the analyte [29,73,75]. From these data the response times are evaluated. It is also possible to estimate how much the signal at a specific time will be different from the signal at the steady state. This information can be used for uncertainty estimation. This uncertainty source can be eliminated almost completely by taking reading when stationary current has been achieved [31].

### 5.6. Linearity

Linearity of response of amperometric sensors has been extensively discussed in the literature [14,16,33–36,38–40,41,59,52,53]. As the mass-transfer rate of the analyte is slow compared to electron transfer, the current is controlled by diffusion rather than the kinetics of the electrode reaction, and this assures a linear dependence of the current over a wide range of concentration [5]. Linearity refers to the analyte concentration range, in which the sensor signal is proportional to concentration. Measurement range is connected with linearity and is defined as the range between the lowest and the highest concentration, which can be determined with assumed accuracy and precision [14].

Accurately prepared calibration mixtures are very important for linearity testing. In the case of gas-phase measurements gas mixtures of controlled composition can be used. Such mixtures are commercially available or can be prepared in the laboratory [32]. In the liquid phase the standard addition method can be used. If accurate preparation of calibration mixtures is difficult then an accurate independent reference method can be used if available. If the linear range of the sensor is established then the sensor should be used in the linear range only. If deviations from linearity occur then these should be corrected for or taken into account by additional uncertainty components [76].

Linearity also depends on the realizable analyte concentrations in the measurement medium. For example solubility of many gases in water is limited and in many cases even at saturation level the responses are still in the linear range. At the same time solubility of e.g., ethanol in water is unlimited and non-linearity can be a problem at higher concentrations. Linearity is assessed at constant conditions varying the analyte concentration in the solution or in the gas phase. Uncertainty due to possible non-linearity can be accounted for by the generic approach as described in reference [76]. The possible non-linearity component of uncertainty is the larger the more the concentrations during calibration and measurement differ.

### 5.7. Zero Current

Zero current (also termed as background current) arises from numerous sources. The most common of them is the oxidation or reduction of electrochemically active impurities and other side reactions [29]. The impurities may be present in the analyzed solution, but as well in the sensor materials. It can be especially important if too high a polarizing voltage is applied. Thus, the concept of zero current is closely related to selectivity [24]. High zero current can also be caused by the analyte from the previous measurements dissolved in the insulating body of the sensor (or in the electrolyte). This is especially noticeable in high-concentration environment. Analyte “stored” this way may slowly diffuse out during future use when the sensor is in an environment where analyte concentration is low [29]. This is similar to the sample carryover effect, frequently observed with different trace analysis techniques.

If the zero current is high then it should be taken into account either in the calibration model (preferably) or as a component of uncertainty. Neither of the two approaches is easy, if the zero current varies and depends on the composition of previous samples. In the former case there will still be an additional uncertainty associated with the inaccuracy of zero current determination. Zero current is determined at the temperature of measurement and zero concentration of the analyte. Under these conditions zero current is the stationary current of the sensor. In the literature only few authors have addressed zero current [22,31,36].

### 5.8. Rounding of the Digital Reading

Modern instruments display results in the digital form, rounding the result and thus introducing uncertainty due to rounding. Whether or not this uncertainty component is of importance is highly dependent on measurement conditions. In most cases its effect is small, but in certain cases can make op to 60% of the uncertainty [31]. Uncertainty due to rounding is easy to take into account: its magnitude is ± 0.5 of the last digit of the reading, with rectangular distribution [77].

### 5.9. Analyte Concentration in Calibration Medium

There is always an uncertainty associated with the analyte concentration in the calibration medium. This uncertainty is transferred to the uncertainty of all measurement results obtained with the sensor, regardless accurate the sensor otherwise is. If reference mixtures are used for calibration then there is usually an accompanying document that contains also the uncertainties of the analyte concentrations. If the calibration mixture is prepared in-house then the uncertainty of analyte concentration is mostly due to gravimetric and/or volumetric measurements and can be estimated using the standard uncertainty estimation approaches [77]. With certain analytes there are additional complications that arise: the analyte may be volatile, prone to decomposition or adsorption, *etc.* These effects significantly complicate preparation of calibration mixtures and evaluating the uncertainty. In some cases calibration mixtures can be prepared only *in situ*. For example, there is up to now no standard solution of dissolved oxygen available [31,45]. Standard substance purity should also be accounted for, which can be difficult, if the impurities do not act as inert compounds but influence the response [78].

### 5.10. Activity of the Analyte and Matrix Effect

Amperometric sensors measure actually not analyte concentration, but activity [29]. Calibrating the sensors in concentration terms implicitly introduces the assumption that the activity coefficient of the analyte is the same during calibration and measurement. This assumption breaks down e.g., when there is a high concentration of salts in the measured medium. In salt-rich water the active concentration of water decreases and this leads to increase of the activity coefficients of most neutral compounds, notably gases (salting-out effect). For example, investigations of the solubility of H_2_S in pure water and NaCl brine solutions at different ionic strengths show significant influence on the activity coefficient of molecular H_2_S [36].

If a calibration made in pure water is used to calculate the concentration of the analyte in real sample with high ionic strength (e.g., sea water) then significant uncertainty is introduced by the difference between the activity coefficient of the analyte in the calibration solutions and the sample during measurement. For example, if in a stream of natural water the dissolved oxygen activity coefficient is 1.075 and the dissolved oxygen concentration as measured by the Winkler method [79] is 4.7 mg dm^−3^ then the reading of an amperometric sensor calibrated in water with negligible ionic strength will be approximately 5.0 mg dm^−3^ [28].

This effect either has to be corrected for or introduced as an additional uncertainty component. This issue has been studied e.g., with dissolved oxygen measurement [28] and it is possible to calculate the activity coefficient of oxygen if the ionic strength is known [29,**Error! Reference source not found.**,^81^]. The activity coefficients found this way are approximate, because conductivity does not directly show the ionic strength of the solution.

### 5.11. Interferences from Other Compounds (Insufficient Selectivity)

The usual assumption in sensor use is that the sensor response is influenced only by the analyte and not by other compounds in the sample matrix. In reality there are often interfering compounds (interferents) that either (1) behave as the analyte or (2) disturb the operation of the sensor in some way.

There are some possibilities to enhance sensor selectivity by sensor design. One of them is choice of the membrane (applicable to GDE and Clark type sensors). Appropriately chosen membrane can efficiently limit the access of interfering substances to the electrode [82]. The second possibility is tuning the composition and pH of the electrolyte solution [2]. The third possibility is to vary the working potential [29] of the sensor. The fourth possibility is to apply filters that trap and eliminate interfering substances [1]. The fifth possibility is to use an auxiliary electrode that works at a lower potential than the working electrode and electrochemically removes the interfering substances [1]. As a general rule lower potential of the working electrode is preferable, because it excludes interference by substances of higher oxidation potential. Nevertheless, selectivity certainly remains an issue with amperometric sensors and is often one of the main uncertainty components in analysis with amperometric sensors. In some drastic cases the sensitivity of the sensor can actually be higher towards an interferent that towards the analyte. For example, this holds in the case of certain ethanol sensors that are more sensitive towards methanol than towards ethanol [33].

In spite of its importance, selectivity has been discussed scarcely in the papers devoted to amperometric sensors. The reason might be that it is extremely difficult to model it, express it numerically or take it into account as an uncertainty source [83]. This is the reason, why most of the discussion on selectivity [16,13,23,24,33,34,38,40–42] remains qualitative: the majority of authors discuss selectivity in yes/no terms only. In a small number of papers also quantitative estimates are given [82,84]. For example, the effect of water on the Au-Nafion® on and Au-ADP SPE electrodes for determination of ethanol and acetaldehyde was investigated. It was found that the signal dramatically increases with humidity content up to approximately 80% r. h., after which further increase does not vary the response considerably. The presence of H_2_O in the gas thus can cause strong interference and has to be either controlled or compensated for. [2] If data are available on sensor selectivity with respect to the potential interferents and if the concentration ranges of the interferents in the samples can be estimated then the uncertainty due to the possible presence of the interferents can be estimated. Alternatively, if it is possible to remove the analyte from the sample and measure the zero current (which will mostly be due to the interferents) then it will be possible to correct the results for the interference. This can be done if the background current is steady and the interfering substances do not contaminate the system [29]. When the background current is large and is not steady, then the only real solution is to remove the interference either before or during measurement using chemical treatment or with some separation device.

## 6. Literature Survey: Conclusions

The literature survey reveals that the main uncertainty sources relevant to amperometric measurement are well known. As a generalization the following “fishbone” diagram of uncertainty sources inherent to amperometric measurements can be presented:

All of the uncertainty sources indicated in Figure 1 have been at least in some context dealt with in the literature. The level of coverage of the uncertainty sources differs very strongly. For example, repeatability as an uncertainty source is almost always included while the activation energy of diffusion is examined only in few works. A potentially very important uncertainty source is interference from other compounds in the matrix. Interference is often mentioned and discussed but rarely handled as an uncertainty source.

Uncertainty sources in amperometric measurements of different analytes are broadly the same. Nevertheless, their magnitudes are strongly dependent on the analyte, the matrix, sensor design and measurement conditions [31]. Therefore it is not to be expected that uncertainty estimation at a reasonable level of rigor can be carried out based purely on literature data.

## 7. Approaches for Uncertainty Estimation in Amperometric Measurement

Most of the available approaches for measurement uncertainty estimation evaluate the uncertainty due to different uncertainty sources and combine these into the combined standard uncertainty *u*_c_ – uncertainty estimate taking into account all significant uncertainty sources and expressed at the level of standard deviation [7,9,70]. For reporting the result usually a higher coverage level is desired than the one provided by *u*_c_ (roughly 68% if the result is Normally distributed) and thus uncertainty is usually reported as expanded uncertainty *U:*

(5) $$U=k\cdot u_{c}$$

where *k* is the coverage factor. Often *k* = 2, which means that the coverage level is roughly 95%, if the result is normally distributed.

The approaches differ in two aspects: (1) how detailed is the examination of the uncertainty sources and (2) how the estimates of the uncertainty sources are combined into *u*_c_. We look at two widespread approaches for uncertainty estimation:

1. Full-fledged modeling approach as proposed in Reference [9]. The use of this approach implies compiling a measurement model for the sensor, in-depth analysis of the uncertainty sources and experiments for quantifying the uncertainty components. This means that the uncertainty is broken down into a number of sources and they are combined using the measurement model. If the output quantity *Y* is found as a function *F* of input quantities *X*_1_ .. *X*_n_ as:

(6) $$Y=F(X_{1},X_{2}...\;X_{n})$$

then the combined standard uncertainty is found as:

(7) $$u_{C}(y)=\sqrt{{\left[\frac{\partial Y}{\partial X_{1}}u(x_{1})\right]}^{2}+{\left[\frac{\partial Y}{\partial X_{2}}u(x_{2})\right]}^{2}+.....+{\left[\frac{\partial Y}{\partial X_{n}}u(x_{n})\right]}^{2}}$$

where *u*(*x*_i_) is the standard uncertainty estimate of *X*_i_. All these standard uncertainty contributions have to be evaluated.

2. Approach based on validation and quality control data. This approach was originally proposed in the handbook published by Nordtest [70] and has later been revisited in reference [7]. The advantages of the approach are that there is no need for a measurement model and in-depth analysis of uncertainty sources. Instead, use can be made of validation data, control charts, reference measurements [71] and participation in interlaboratory comparisons–all of which are rather accessible even to routine analysis laboratories. The different uncertainty sources are accounted for by two principal components–*u*(*R*_W_) accounting for all the random factors (at lab level) contributing to the uncertainty and *u*(*bias*) accounting for all the systematic factors (at lab level) contributing to the uncertainty. These two are combined as follows:

(8) $$u_{c}=\sqrt{u{\left(R_{w}\right)}^{2}+u{\left(\mathrm{bias}\right)}^{2}}$$

Reference [70] envisages the ways to estimate these two components by using control charts [85] (for *u*(*R*_W_)), analysis of certified reference materials or participation to interlaboratory comparisons (for *u*(*bias*)). The uncertainty component accounting for possible bias is found as follows [86]:

(9) $$u(\mathrm{bias})=\sqrt{{\mathrm{RMS}}_{\text{bias}}^{2}+u{\left(C_{\text{ref}}\right)}^{2}}$$

where *RMS*_bias_ is the root mean square of differences found as:

(10) $${\mathrm{RMS}}_{\text{bias}}=\sqrt{\frac{\Delta_{1}^{2}+\Delta_{2}^{2}+...+\Delta_{n}^{2}}{n}}$$

where Δ_i_ are the differences between the results of the procedure and *C*_ref_ and *u*(*C*_ref_) is the standard uncertainty of *C*_ref_. Contrary to the model-based approach the uncertainty estimate obtained with the Nordtest approach does not characterize a single measurement result but rather gives an average uncertainty value obtained in a laboratory using a given measurement procedure [7]. This estimate is appropriate for the measurement conditions under which the uncertainty components *u*(*R*_w_) and *u*(*bias*) were evaluated. Further comments on the approaches are given in the case studies presented below.

### 7.1. Case study 1: Model-Based Measurement Uncertainty Estimation

Model-based measurement uncertainty estimation [9] closely follows the operation principle of the sensor, expressed by the measurement model. An adequately compiled model allows taking into account all significant uncertainty sources [77]. The uncertainty estimate obtained with the model-based approach are directly relevant to a particular measurement situation and allows to take into account all the aspects of the measurement, such as temperature, calibration conditions, sensor design, *etc*. [31]. The outcome of uncertainty estimation with this approach is not only the uncertainty estimate but also the uncertainty budget, which allows seeing where most of the uncertainty comes from and thus provides valuable information on improving the measurement. This is certainly a big advantage of this approach. At the same time, in order to get a realistic uncertainty estimate, all the individual uncertainty components have to be discovered and also quantified. This requires careful investigation of the measurement procedure and high level of competence.

We present here a case study of uncertainty estimation of amperometric dissolved oxygen measurement. It was originally published in reference [31] and full details can be found there. This case study is useful for demonstrating how much measurement conditions can affect uncertainty.

The WTW OXI340i analyzer with a CellOx 325 sensor was used for measurements. The calibration and measurement conditions correspond to cases 1 and 4 in reference [31] and have been described in detail there. Case 1 represents a laboratory measurement under nearly ideal conditions. The sensor membrane and electrolyte have been freshly changed. Calibration has been carried out in water immediately before measurement. The stirring speed is the same during measurement and calibration and is quite high: 30 cm s^−1^. Case 4 represents a typical measurement situation in a laboratory doing field work. Sensor’s membrane is 0.5 months old. Calibration was carried out in laboratory at 20 °C (stirring speed 20 cm s^−1^) 5 days before measurement. The measurement is performed under outdoor conditions with different water temperatures. The estimated stirring speed during measurement is 10 cm s^−1^ (this is a suitable estimate of the flow speed in the case of slow river flow or moving the sensor up and down during measurement). In both cases saturated oxygen concentrations are examined at various temperatures. The results are presented in Table 2.

The uncertainty budgets for both cases at temperature 5 and 20 °C are presented in Table 3.

At all temperatures (except in the case with zero oxygen concentration) the uncertainty is significantly lower under laboratory conditions, because calibration was carried out immediately before measurement and the stirring speed is equal during calibration and measurement and the sensor membrane has been freshly changed. As can be easily seen from Table 3, under different measurement conditions different uncertainty sources have the most significant contributions.

At 20 °C in Case 1 the contribution of the calibration solution concentration uncertainty is near 50%, meaning that these measurement conditions allow to obtain accuracy approaching the highest possible with that sensor–calibration solution concentration is a factor extraneous to the sensor. At 5 °C in Case 1 the uncertainty is mainly due to the quite strong temperature correction –the activation energy of permeation of oxygen through the membrane *E*_sme_membrane_ is the main uncertainty source. In Case 4 at both temperatures the main part of uncertainty comes from the uncertainty of the stirring speed. The reason is that this particular sensor has a very thin membrane with high O_2_ permeability and under field conditions the stirring speed can only be vaguely estimated.

In order to use this approach successfully the uncertainty contributions due to all these (and other) factors have to be estimated reliably. Detailed discussion on these (and some more, corresponding to different concentrations) uncertainty budgets can be found in reference [31].

### 7.2. Case study 2: Measurement Uncertainty Estimation Based on the Nordtest [70] Approach

The same instrument was used as in Case study 1. However, due to the nature of the Nordtest approach–pooling of the data over a long time period–the sensor properties–age of the membrane and time that has passed from the last calibration –cannot be as well defined as in the case of the ISO GUM modeling approach. The approach rather evaluates an *average* uncertainty of the measurement procedure as applied under the normal working conditions of a particular laboratory.

The component taking into account random effects *u*(*R*_w_) was estimated from a control chart made at saturation concentration at temperature 20 °C and from these data *u*(*R*_w_) = 0.089 mg/L. In order to be applicable also to concentrations lower than saturation we use this estimate as relative standard uncertainty *u*_rel_(*R*_w_) = 0.98%. Due to the working principle of amperometric dissolved oxygen measurement it can be assumed that this relative uncertainty estimate is in broad terms constant over the different concentrations [31].

The *u*(*bias*) component can be estimated from the results of interlaboratory comparison measurements from references 0 and 0 as well as from reference measurements with air-saturated water at different temperatures. The bias estimates obtained are presented in Table 4:

The uncertainty estimates obtained from these data using eqs 8–10 are given in Table 5. The uncertainty of the reference value at the saturation conditions was 0.15 mg/L (*k* = 2). The uncertainty of the reference value of zero solution was 0.01 mg/l (*k* = 2) [88].

In this case both control charts and interlaboratory comparisons were carried out at saturation concentrations. Therefore these uncertainty estimates are more suitable for higher concentrations.

### 7.3. Comparison of the Model- and Nordtest Based Uncertainty Estimation Approaches

**Figure 2.**
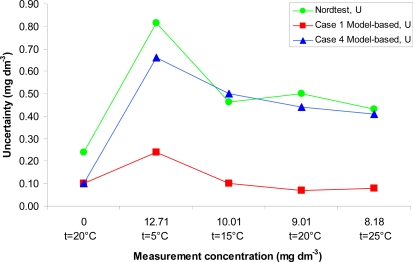
Expanded uncertainties for all conditions using two estimation approaches.

Comparison of the uncertainty estimates obtained with both approaches is presented in Figure 2. As seen from the figure the uncertainties obtained using the Nordtest approach and the ISO GUM approach under the non-ideal conditions (Case 4) agree well. This is remarkable, given the completely different foundations of the data used for uncertainty evaluation. It is fair to say that in practice this level of agreement between the ISO GUM modeling and Nordtest approaches is not always found. The uncertainty under nearly ideal laboratory conditions is expectedly significantly lower – up to five times–than in the remaining two cases.

The ISO GUM approach allows evaluating uncertainty for a particular measurement result taking account the particular measurement conditions. In contrast, the Nordtest approach allows evaluating an averaged uncertainty, which takes into account the average state of the equipment and working practices over a long period of time.

Mathematically the Nordtest approach is simpler, but availability of data for a sufficiently long period of time is necessary. The most important issue with the Nordtest approach is estimation of the bias. It may often be difficult to find reference values of sufficiently high quality for that. The most accessible reference values for laboratories are generally results of Interlaboratory comparison measurements. However, their reference values can be of low quality, especially if consensus values based on participant results are used. For example, in 1981 an interlaboratory comparison measurement of dissolved oxygen concentration was carried out [89] at two concentrations 1.20 and 5.86 mg dm^−3^. The mean absolute difference of the participant results from the reference values was 0.6 mg dm^−3^. Also, 11 laboratories out of 14 obtained higher results than the first reference value and all laboratories obtained results higher than the second reference value.

## 8. Practical Notes on Achieving Accuracy When Measuring with Amperometric Sensors

The lowest uncertainty is obtained when the analyte concentration is near the calibration concentration(s), measurement is carried out during a short time after calibration, measurement and calibration are carried out in the same medium and the flow rate and temperature are the same during measurement and calibration. It must be made sure that the stationary current has indeed been reached. Strong interferences should be eliminated if present and salinity correction carried out (if relevant). When measuring low analyte concentrations the zero current has to be either corrected for or taken into account as uncertainty source. If so done the main uncertainty sources are those associated with analyte concentration in calibration solutions and repeatability/stability of the sensor (in the medium concentration range) or zero current (in the low concentration range).

## 9. Conclusions

A large number of different factors cause uncertainty in analysis using amperometric sensors. All of them have been addressed in the literature and estimates of the uncertainty invoked by them can be found in the literature. However, different uncertainty sources differ vastly by their coverage and only a handful of papers describe calculation of combined standard uncertainty that takes into account all relevant uncertainty sources.

Uncertainty estimation by the modeling approach, which explicitly takes into account all major uncertainty sources and combines them using a measurement model, needs a high level of knowledge about the measurement procedure. The alternative–Nordtest approach–is less demanding on the detail of knowledge but needs ample validation data. The approaches yield uncertainties that refer to different situations–the particular measurement under question and the measurement procedure in routine use at the laboratory, respectively.

The case studies demonstrate that even with the same sensor the relative contribution of the different uncertainty sources can be very different depending on the sample, condition of the sensor and measurement conditions.

## Figures and Tables

**Table 1.** Uncertainty sources of amperometric sensors discussed in the literature.

**Analyte**	**Phase**	**Response time**	**Linearity***^a^*	**Repeatability**	**Drift**	**Interferences**	**Ref**.
O_2_	Gas	<20 s^b^	Nominal Range: 0–2 ppm, linearity: linear		<5% signal loss/year		[49]
O_2_	Gas	20 s*^c^*	Range: 0–25%, linearity: ± 1% of full scale	± 0.1% of range	± 0.25% O2 per week		[50]
O_2_	Gas	General purpose: 180 s*^c^* Fast Response: 30 s*^c^*	0.05–60 mg dm^3^		<1% per month		[51]
H_2_	Gas	<70 s*^c^*	Nominal Range: 0–10,000 ppm, linearity: linear	2% of signal	<2% signal loss/month	CO; H_2_S; NO; HCN; C_2_H_4_	[52]
H_2_	Gas	Ppm level: 20–50 s % level: 5–20 s	0.2–2%	± 10% *^d^*			[53]
SO_2_	Gas	≤15 s*^c^*	Nominal Range: 0–20 ppm, Output linearity: linear	2% of signal	<2% signal loss/month	CO; H_2_S; NO_2_; HCN; Cl_2_; HCl	[54]
O_3_	Liquid	<90 s*^c^*	0.005–2 ppm	1.0%	Calibration interval 2 months	No interference from Br_2_, chloramines, Cl_2_, ClO_2_ or H_2_O_2_	[55]
NO	Gas	≤15 s*^c^*	Nominal Range: 0–100 ppm, Output linearity: linear	2% of signal	<2% signal loss/month	H_2_S; HCl; NO_2_	[56]
NH_3_	Gas	<90 s*^c^*	Measuring range: 0–1000 ppm, Linearity: <5% full scale		<10% per 6 months	H_2_S; SO_2_	[57]
HCHO	Liquid	Tens of seconds	0.002–1.25 mg mL^−1^		Ca 1.4% per hour	CH_3_OH; HCHO; HOCH_2_CH_2_OH	[58]
H_2_S	Liquid	<100 ms	2–300 μM	2.5%	<5% per day	SO_2_; CH_3_CH_2_SH	[36]
CO	Gas	7 s	0.70–56 μg mL^−1^	5.3% (n = 5)			[35]
SO_2_	Liquid	4 s	4·10^−7^–1·10^−3^ mol dm^−3^	± 3%		H_2_S; NO	[16]
SO_2_	Gas	1 s	8·10^−9^–2·10^−4^ mol dm^−3^	± 3%		H_2_S; NO	[16]
SO_2_	Gas	189 s*^c^*	5–500 ppm*^e^*			None*^f^*	[41]
H_2_S	Gas	Ca. 10 s	0–100 ppm	± 1 ppm*^g^*			[59]
PH_3_	Gas	4.6 s*^c^*	0–100 ppm	± 3%*^g^*	Good long-term stability		[60]

^a^ Linearity data has been presented the way it was given in the original paper.

^b^ T_95_

^c^ T_90_

^d^ Reproducibility, the stability of the sensor was monitored for a period of three weeks and found to be stable within ±10% of the concentration value.

^e^ The linear equation was y = −0.07x – 2.66 with a correlation coefficient of 0.9946.

^f^ Other copresent gases, such as CO, NO, NH_3_ and CO2, did not cause interference under these conditions.

^g^ Reproducibility

**Table 2.** The expanded uncertainties (*k* = 2) of WTW OXI340i with a CellOx 325 sensor for laboratory and field conditions by model-based measurement uncertainty estimation.

***t*_meas_ (°C)**	***C*_meas_ (mg/L)**	***U* (mg/L)**	***U* relative (%)**	***U* (mg/L)**	***U* relative (%)**
		*Case 1 laboratory conditions*		*Case 4 field conditions*	
20	0	0.10	-	0.10	-
5	12.71	0.24	1.9%	0.66	5.2%
15	10.01	0.10	1.0%	0.50	5.0%
20	9.01	0.07	0.8%	0.44	4.9%
25	8.18	0.08	0.9%	0.41	5.0%

**Table 3.** Uncertainty budgets for cases 1 and 4 at temperatures of 5 and 20 °C.

**Inputs***^a^*	**Calibration environment: water**			

	**Case 1**	**Case 4**	**Case 1**	**Case 4**

	**Measurement conditions**			

*C*_meas_ (mg dm^−3^)	12.71	12.71	9.01	9.01
*t*_meas_ (°C) ^a^	5	5	20	20
stirring speed_meas (cm s^−1^)	30	30	30	30
*t*_cal_ (°C) ^a^	20	20	20	20
u(*p*_cal_) (Pa)	150	150	150	150
stirring speed__cal_ (cm s^−1^)	30	20	30	20
Δday_new_Δcal-meas_ (day)	0	5	0	5
Δday_old_Δcal-meas_ (day)	0	0	0	0
Δmonth (month)	0	0.5	0	0.5

**Input Parameters (*x*_i_)***^a^*	**Uncertainty contributions (indexes) of the input parameters *x*_i_**			

*t*_cal_	0%	0%	0%	0%
Δ*T*_instab_	0%	0%	1%	0%
Δ*J*_0_	2%	0%	0%	0%
Δ*J*_cal_output_	2%	1%	**12%**	1%
*p*_cal_	3%	0%	**15%**	0%
Δ*C*_sat_cal_	**9%**	1%	**47%**	1%
Δ*p*_CO2_	0%	0%	1%	0%
Δ*p*_H2O_cal_	2%	0%	9%	0%
Δ*C*_read_cal_	0%	0%	0%	0%
*t*_meas_	3%	0%	0%	0%
Δ*J*__meas_output_	2%	3%	**12%**	4%
Δ*C*_read_meas_	0%	0%	1%	0%
Δ*l*_sme_drift_	0%	2%	4%	2%
Δ*E*_sme_drift_	0%	0%	0%	0%
Δ*J*_stir_	0%	**81%**	0%	**91%**
*E*_sme_membrane_	**77%**	**10%**	0%	0%

	**Expanded uncertainties (*k* = 2) of *C*_meas_**			

*U*(*C*_meas_)	0.24	0.66	0.07	0.44
*U(C*_meas_), relative	1.9%	5.2%	0.8%	4.9%

^a^ The definitions of all the quantities and parameters are given in reference [31].

**Table 4.** Bias estimates obtained from the reference measurements and interlaboratory comparisons.

***t*_meas_ (°C)**	**16.01.2006***^a^*	**7.03.2006***^b^*	**9.06.2006***^a^*

0 mg/L	0.04	0.20	0.04
5	−0.35	−0.56	0.01
15	−0.28	−0.18	0.07
20	−0.32	−0.11	−0.18
25	−0.31	−0.03	−0.08

^a^ Reference measurements.

^b^ Interlaboratory comparisons.

**Table 5.** The uncertainties of WTW OXI340i with a CellOx 325 sensor by the Nordtest approach.

***t*_meas_ (°C)**	***C*_meas_ (mg dm^−3^)**	***RMS*_bias_ (mg dm^−3^)**	***u*(bias) (mg dm^−3^)**	***U* (mg dm^−3^)**	***U* (%)**

20	0	0.12	0.12	0.24	
5	12.71	0.38	0.39	0.82	6.4
15	10.01	0.20	0.21	0.46	4.6
20	9.01	0.22	0.23	0.50	5.5
25	8.18	0.19	0.20	0.43	5.3
